# H-index, mentoring-index, highly-cited and highly-accessed: how to evaluate scientists?

**DOI:** 10.1186/1742-4690-5-106

**Published:** 2008-11-25

**Authors:** Kuan-Teh Jeang

**Affiliations:** 1 The National Institutes of Health, Bethesda, MD, USA

## Abstract

How best to evaluate scientists within a peer group is a difficult task. This editorial discusses the use of the H-index and total citations. It also raises the consideration of a mentoring-index and the value of understanding the frequency that a published paper is accessed by readers.

## Editorial

### Key performance indicators

A challenging question in peer-reviewed science is how to distribute judiciously resources amongst a large number of competing researchers. What are the "key performance indicators" that should be used to evaluate scientists who pursue similar research interests? One popular discussion is to ask how many times a person has published articles in journals with a high impact factor (IF). Several "quirks" in the way that a journal's IF is calculated have prompted many individuals to question whether this number reliably reflects the citation frequency of research articles that are published in the journal [1]. Recently, a scientist's H-index (HI) [2] has been suggested as a more informative measure of his/her scientific productivity [1].

### H-index and total citations

The predictive value of the HI does have limitations [3]. However, in a 2007 survey of *Retrovirology *editorial board members, it was noted that an individual's H-number correlated well with the absolute frequency that his/her published papers were cited in the scientific literature [1]. A mid-October 2008 update of the 2007 survey, using numbers from the Scopus database , continues to support this correlation (Table 1). Thus, within a well-delimited field of research, a scientist's HI and his/her total citations appear to be reasonably quantitative peer-measures, seemingly superior to the colloquial banters about "high impact" papers. It should be noted that different databases measure HI numbers over varying time periods, and are not directly comparable. In general, a HI number increases with the length of time over which it is measured; hence, older scientists would usually be expected to sport HI numbers higher than their younger counterparts

**Table 1:** H-index and citation frequencies of selected *Retrovirology *editorial board members.

**Title**	**Name**	**Role within *Retrovirology***	**Institution**	**City**	**Country**	**H index**	**Total times cited since 1996**
Dr.	Kuan-Teh Jeang	Editor-in-Chief	NIH	Bethesda	USA	43	9082

Dr.	Monsef Benkirane	Editor	CNRS	Montpellier	France	20	1751

Dr.	Ben Berkhout	Editor	Academic Med. Ctr	Amsterdam	the Netherlands	38	6022

Dr.	Andrew ML Lever	Editor	Cambridge University	Cambridge	UK	19	1919

Dr.	Mark Wainberg	Editor	McGill University	Montreal	Canada	39	9519

Dr.	Masahiro Fujii	Editor	Niigata University	Niigata	Japan	19	1686

Dr.	Michael Lairmore	Editor	Ohio State University	Columbus	USA	20	1933

Dr.	Michael Bukrinsky	Ed Board	George Washington Univ	Washington DC	USA	25	4913

Dr.	Dong-yan Jin	Ed Board	Hong Kong U	Hong Kong	China	22	2402

Dr.	Klaus Strebel	Ed Board	NIH	Bethesda	USA	25	3889

Dr.	Tom J. Hope	Ed Board	U. Illinois	Chicago	USA	26	4307

Dr.	Ariberto Fassati	Ed Board	University College	London	England	11	524

Dr.	Stephane Emiliani	Ed Board	Cochin Institute	Paris	France	17	1774

Dr.	Patrick Green	Ed Board	Ohio State	Columbus	USA	17	918

Dr.	Mauro Giacca	Ed Board	Int. Ctr. Genetics	Trieste	Italy	35	5051

Dr.	Olivier Schwartz	Ed Board	Institut Pasteur	Paris	France	27	3657

Dr.	Leonid Margolis	Ed Board	National Inst Child Health	Bethesda	USA	22	1745

Dr.	Fatah Kashanchi	Ed Board	George Washington U.	Washington DC	USA	26	2503

Dr.	Masao Matsuoka	Ed Board	Kyoto University	Kyoto	Japan	24	1992

Dr.	Naoki Mori	Ed Board	University of the Ryukyus	Okinawa	Japan	24	1982

Dr.	Chou-Zen Giam	Ed Board	Uniform Services Med School	Bethesda	USA	14	1454

Dr.	David Derse	Ed Board	NCI	Frederick	USA	13	1667

Dr.	Tatsuo Shioda	Ed Board	Osaka Univ	Osaka	Japan	24	1956

Dr.	John Semmes	Ed Board	Eastern Virginia Med College	Norfolk	USA	27	2953

Dr.	Anne Gatignol	Ed Board	McGill Univ.	Montreal	Canada	14	1012

Dr.	Rogier Sanders	Ed Board	Academic Med Ctr.	Amsterdam	the Netherlands	13	845

Dr.	Chen Liang	Ed Board	McGill Univ.	Montreal	Canada	19	915

Dr.	Finn Skou Pedersen	Ed Board	University of Aarhus	Aarhus	Denmark	19	1490

Dr.	Janice Clements	Ed Board	Johns Hopkins Med School	Baltimore	USA	23	3454

Dr.	Renaud Mahieux	Ed Board	Pasteur Inst	Paris	France	23	1312

Dr.	Chris Aiken	Ed Board	Vanderbilt University	Nashville	USA	18	2347

Dr.	Neil Almond	Ed Board	NIBSC	Potters Bar	UK	12	1121

Dr.	Stephen P. Goff	Ed Board	Columbia University	New York	USA	41	13771

Dr.	Johnson Mak	Ed Board	Burnet Inst. Med. Research	Victoria	Australia	15	1298

Dr.	Christine Kozak	Ed Board	NIH	Bethesda	USA	29	7489

Dr.	Greg Towers	Ed Board	University College	London	UK	17	1392

Dr.	Graham Taylor	Ed Board	Imperial College	London	UK	15	1567

Dr.	Eric Cohen	Ed Board	Univ. Montreal	Montreal	Canada	27	3221

Dr.	William Hall	Ed Board	University College Dublin	Dublin	Ireland	21	2071

Dr.	Warner Greene	Ed Board	UCSF	San Francisco	USA	39	10133

Dr.	Jean-luc Darlix	Ed Board	U. Lyon	Lyon	France	32	5654

Dr.	Axel Rethwilm	Ed Board	U. Wuerzburg	Wuerzburg	Germany	22	2040

Dr.	Eric Freed	Ed Board	NCI	Frederick	USA	29	4415

Dr.	Toshiki Watanabe	Ed Board	Univ. of Tokyo	Tokyo	Japan	22	2167

Dr.	Mari Kannagi	Ed Board	Tokyo Med and Dental U	Tokyo	Japan	15	1350

Dr.	Frank Kirchhoff	Ed Board	University of Ulm	Ulm	Germany	30	4520

Dr.	Jennifer Nyborg	Ed Board	Colorado State U	Fort Collins	USA	17	1571

Dr.	Akifumi Takaori-Kondo	Ed Board	Kyoto University	Kyoto	Japan	13	589

Dr.	Marc Sitbon	Ed Board	CNRS	Montpellier	France	12	690

Dr.	Paul Gorry	Ed Board	MacFarlane Burnet Institute	Melbourne	Australia	13	607

Dr.	David Harrich	Ed Board	Queensland Inst Medical Res.	Brisbane	Australia	12	1000

Dr.	Susan Marriott	Ed Board	Baylor	Houston	USA	14	1021

Dr.	Damian Purcell	Ed Board	U Melbourne	Melbourne	Australia	12	902

Dr.	Alan Cochrane	Ed Board	U Toronto	Toronto	Canada	10	1080

Dr.	Yiming Shao	Ed Board	China CDC	Beijing	China	13	977

Dr.	Vinayaka Prasad	Ed Board	Albert Einstein College Medicine	New York	USA	18	1187

### A time for a mentoring-index?

Scientists do research and also mentor younger colleagues. Good mentoring should be a significant consideration of one's contribution to science. The HI might measure research productivity, but currently there does not appear to be a "mentoring index" (MI). Accepting that mentoring is an important component of a scientist's career, one could propose to construct a MI derived as a composite value based on the current HI of trainees during an earlier period with a given mentor. For example, a MI for scientist X reflecting his/her mentoring influence during the 1991 to 1995 period could be calculated from the sum of today's HI for all the first authors from his/her laboratory on papers published during 1991 to 1995 with scientist X as the last author. As an example, for Kuan-Teh Jeang (KTJ) during the 1991–1995 period, there were eight different first authors who listed the same laboratory affiliation as KTJ and who published papers with KTJ as the last author. The eight individuals, (with current HI in parentheses) A. Gatignol (14), B. Berkhout (38), B. Dropulic (9). O.J. Semmes (27), Y.N. Chang (5), F. Majone (5), A. Joshi (2) and L.M. Huang (19), provide a total HI of 14 + 38 + 9 + 27 + 5 + 5 + 2 + 19 = 119. If one divides 119 by 8, a MI of 14.8 for KTJ is derived. This number could be used for comparing KTJ to others for mentoring contributions during a defined period (e.g. 1991 to 1995). Of course, comparisons are meaningful only when done amongst appropriate peer groups. A focus on using the HI of previous trainees in evaluating established scientists could encourage the development of long-lasting mentoring relationships that continue even after the trainees have departed the mentors' laboratories.

### Frequency of citation versus frequency of access

The above discussions of HI, MI, citation frequencies, and impact factor presume the primacy of citations as a measure of scientific value. What if this presumption is off-the-mark? Is there another value that could be considered? In other areas of communication (book publishing, music distribution) where citation metrics are irrelevant, the numbers of readers (copies of books sold) and listeners (number of albums sold or songs downloaded) are used to gauge impact. In the modern internet era, the frequency of "hits" or accesses to portals such as YouTube or Facebook quantitatively gauges relative importance. In this respect, should the frequency of accesses to online Open Access scientific articles similarly matter? To begin to explore this question, I examined the top 15 "all time" most highly accessed papers at *Retrovirology *. In this dataset, four 2006 papers (excluding a meeting report, [4]) were identified that have been accessed 23,634; 8,592; 8,304; and 7,902 times respectively [5], [6], [7], [8]. These four highly accessed papers have been cited to date 14, 13, 15, and 14 times, placing them in the top 15% of cited *Retrovirology *papers published in 2006. On the other hand, the four *Retrovirology *papers published during 2006 that are currently the most frequently cited [9], [10], [11], [12] (cited 27, 23, 21, 20 times) are not the four which are the most highly accessed. Thus, high readership does seem to produce high citation frequency, but high citation frequency does not always require high readership. This pattern suggests that Open Access readers encompass those who simply read and those who read and also write papers that cite other papers. Citation numbers measure the latter group, while access numbers measure both groups. Arguably, it is unclear that a published paper's influence on one group (citations) counts while the less well-tabulated impact on the second group (accesses) counts not. The relative merits of citations versus accesses require further validation.

### Acknowledgements

I thank Mark Wainberg, Andrew Lever, and Ben Berkhout for critical readings of this editorial. The values shown in Table 1 are to be viewed as illustrative examples and are not to be regarded as fully accurate. The views expressed are the author's personal opinion and do not represent the position of the author's employer, the National Institutes of Health, USA. Research in KTJ's laboratory is supported by NIAID Intramural funds. I thank Christina Bezon for assistance with Table 1.

## Competing interests

The author declares that he has no competing interests.
